# Aggregation-induced emission effect on turn-off fluorescent switching of a photochromic diarylethene

**DOI:** 10.3762/bjoc.15.217

**Published:** 2019-09-20

**Authors:** Luna Kono, Yuma Nakagawa, Ayako Fujimoto, Ryo Nishimura, Yohei Hattori, Toshiki Mutai, Nobuhiro Yasuda, Kenichi Koizumi, Satoshi Yokojima, Shinichiro Nakamura, Kingo Uchida

**Affiliations:** 1 Department of Materials Chemistry, Ryukoku University, Seta, Otsu, Shiga 520-2194, Japan; 2 Department of Materials and Environmental Science, the University of Tokyo, 4-6-1, Komaba, Meguro-ku, Tokyo 153-8505, Japan; 3 Japan Synchrotron Radiation Research Institute, 1-1-1 Kouto, Sayo-cho, Sayo-gun, Hyogo 679-5198 Japan; 4 Nakamura Laboratory, RIKEN Cluster for Science, Technology and Innovation Hub, 2-1 Hirosawa, Wako, Saitama 351-0198, Japan; 5 Tokyo University of Pharmacy and Life Sciences, 1432-1 Horinouchi, Hachioji, Tokyo 192-0392, Japan

**Keywords:** AIE, diarylethene, ESIPT fluorescent switching, turn-off fluorescence

## Abstract

**Background:** Diarylethenes are well-known photochromic compounds, which undergo cyclization and cycloreversion reactions between open- and closed-ring isomers. Recently, diarylethene derivatives with photoswitchable fluorescent properties were prepared. They are applicable for fluorescence imaging including bio-imaging. On the other hand, a new system called “excited state intramolecular proton transfer (ESIPT)” is reported. In the system, absorption and emission bands are largely separated due to the proton transfer, hence it showed strong fluorescence even in the crystalline state. We aimed to construct the photochromic system incorporating the ESIPT mechanism.

**Results:** A diarylethene incorporating a fluorescent moiety that exhibit ESIPT behavior was prepared. The ESIPT is one of the examples which express the mechanisms of aggregation-induced emission (AIE). This compound emits orange fluorescence with a large Stokes shift derived from ESIPT in aprotic solvents such as THF or hexane, while it exhibits only a photochromic reaction in protic solvents such as methanol. In addition, it shows turn-off type fluorescence switching in an aprotic solvent and in crystals. The fluorescence is quenched as the content of closed-ring isomers increases upon UV light irradiation.

**Conclusions:** A diarylethene containing an ESIPT functional group was prepared. It showed fluorescent turn-off behavior during photochromism in aprotic solvents as well as in crystalline state upon UV light irradiation. Furthermore, it showed AIE in THF/water mixtures with blue-shift of the emission.

## Introduction

Diarylethenes are well-known photochromic molecules and are widely applied to molecular switches and systems [1–2]. Recently, the photo-switching of ﬂuorescence signals by using diarylethene switches has attracted much attention because of the potential in various optoelectronic applications [3]. For example, fluorescence switching for molecular level recording [4], multiple-fluorescence for logic circuits [5] as well as biological applications [6] and super-resolution microscopy for bio-imaging [7–8]. The fluorescence switching in solid state is attracting much attention from both scientific and technological points of view, such as sensors, electroluminescent displays, memory devices [9].

On the other hand, molecular aggregation also affects the intensity of fluorescence [10–11]. Some material shows fluorescence by aggregation (aggregation-induced emission (AIE)), while the others decay the fluorescence by aggregation (aggregation-caused quenching (ACQ)). The luminogenic materials with AIE have attracted much interest since Tang et al. reported the AIE concept [12]. The introduction of photo-switching ability in the system will be interesting for creating new AIE systems. In addition, organic photochromic crystals are inherently capable of photo-reversible luminescence switching because the electronic structures of photochromic molecules reversibly change upon photoisomerization [13–14]. However, such a fluorescent system in condensed phase emits fluorescence often absorbed in adjacent molecules, therefore a large Stokes shift is indispensable for such a system.

One of the possible choices to achieve such a large Stokes shift is to introduce the excited-state intramolecular proton transfer (ESIPT). The process of ESIPT is a fast process even comparable to the internal conversion [15–16]. This is because the proton transfer occurs through an intramolecular hydrogen bond. This is also an origin of a large Stokes shift (8,000–11,000 cm^−1^) in the emission from the ESIPT state. Consequently, yellow luminescence was observed by UV-excitation [17–18]. Mutai et al. reported an ESIPT luminescence of an imidazo[1,2-*a*]pyridine derivative, in which remarkable fluorescence was observed and no overlapping from the absorption band due to the large Stokes shift of the fluorescence [19–20]. Photochromic diarylethene systems with ESIPT moieties are already reported in some research groups [21–23].

Herein, we prepared a diarylethene incorporating the imidazo[1,2-*a*]pyridine moiety with ESIPT ability and reported the fluorescence switching of the system.

## Results and Discussion

**Figure 1:**
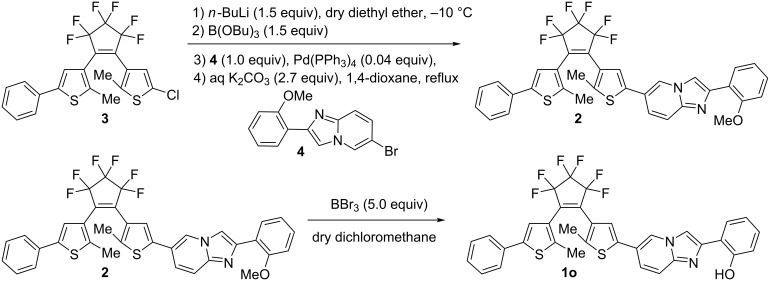
Synthetic procedure for a diarylethene (**1o**).

The diarylethene **1o** having an ESIPT moiety was prepared by a coupling reaction of asymmetric diarylethene **3** [24] and 6-bromo-2-(2’-methoxyphenyl)imidazo[1,2-*a*]pyridine **4** [25] followed by ether cleavage according to Figure 1.

**Figure 2:**
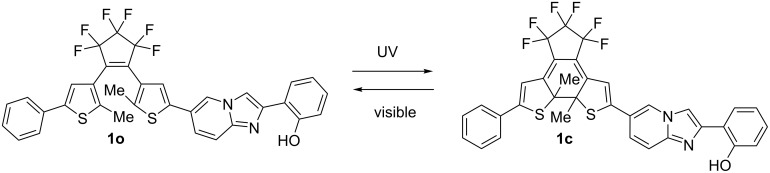
Photochromic reaction of diarylethene **1o** having an ESIPT moiety.

**Figure 3:**
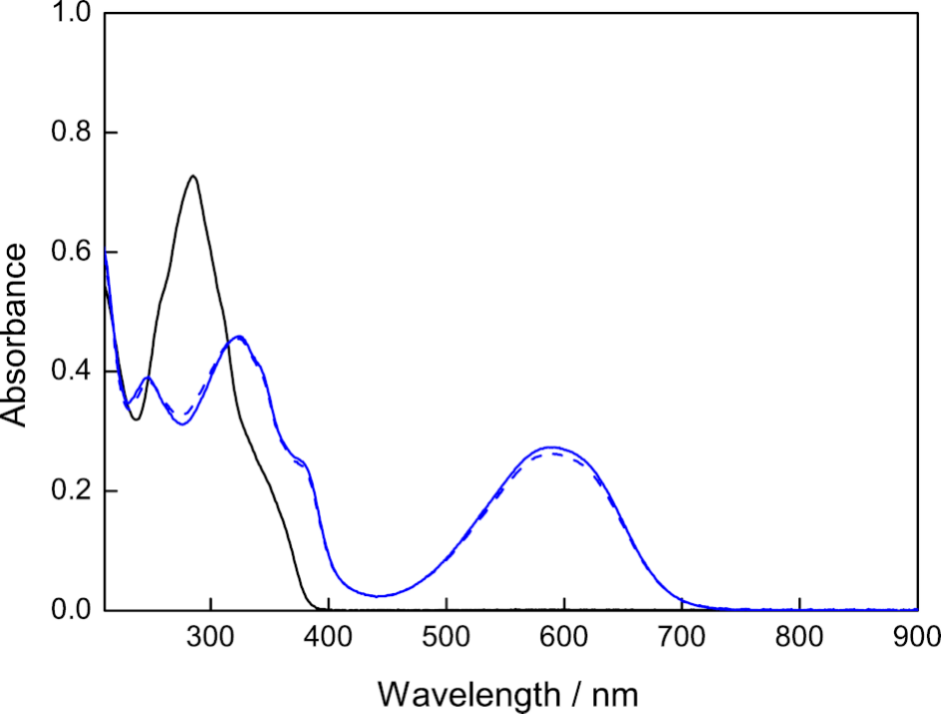
Absorption spectral changes of diarylethene **1o** having an ESIPT moiety in THF (*c* = 1.3×10^-5^ M). Black solid line: **1o**, blue solid line: **1c**, blue broken line: photostationary state under irradiation with 313 nm light (**1o**/**1c** = 4.2: 95.8) (irradiation. for 30 s).

The photochromic reaction and spectral changes are shown in Figure 2 and Figure 3. The photochromic absorption spectral changes in THF are shown in Figure 3. Upon UV light irradiation of the solution of **1o**, the color changed to blue with increasing the absorption band at 587 nm of the closed-ring isomer **1c**, then by visible light irradiation the color disappeared and reproducing the absorption spectra at 285 nm of **1o**. The cyclization and cycloreversion reactions of **1** were measured in THF and methanol. The results were summarized in Table 1.

**Table 1:** Photochromic properties of a diarylethene **1** in methanol and THF.

compounds	λ_max_/nm (ε/10^4^ M^−1^ cm^−1^)		Φ_o→c_ (313 nm)	Φ_c→o_ (577 nm)
	open-ring isomer	closed-ring isomer		

**1** (in THF)	285 (5.7)	587 (2.1)	0.31	0.0062
**1** (in methanol)	283 (5.5)	584 (2.0)	0.34	0.0073

The quantum yields of cyclization and cycloreversion reactions of **1o** in THF are obtained to be 0.31 and 6.2 × 10^−3^, respectively. They are a little bit smaller than those of simple diarylethene switches having thiophene rings as aryl groups [26]. It may be due to the connection with an ESIPT moiety. Only slight changes were observed in absorption spectra as well as photochromic quantum yields in two solvents.

**Figure 4:**
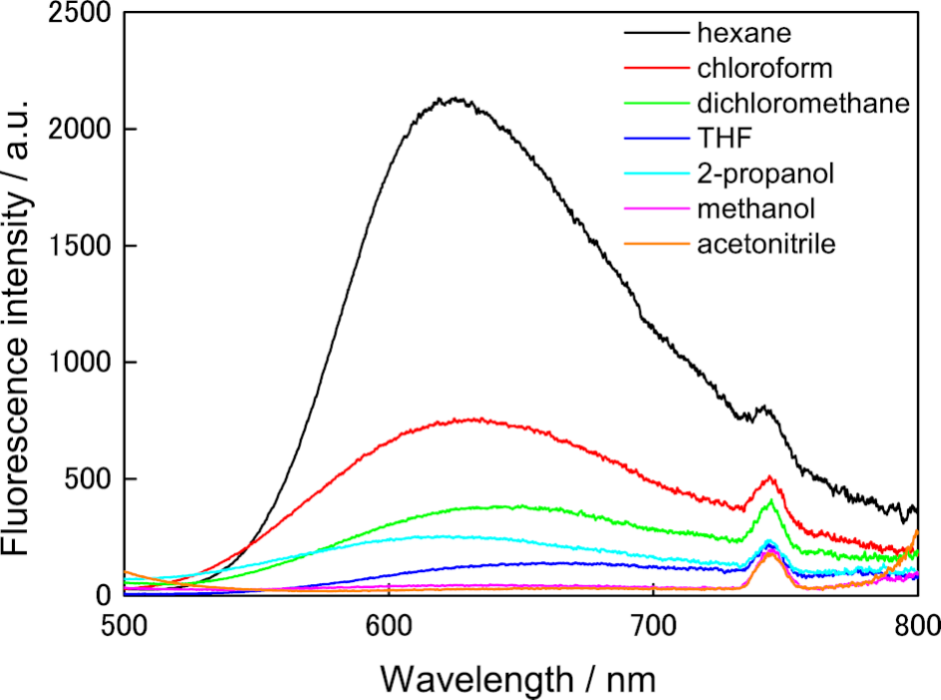
Fluorescent spectra of **1o** in several solvents (λ_ex_ = 370 nm). Hexane (black line), chloroform (red line), dichloromethane (green line), THF (blue line), 2-propanol (pale blue line), methanol (pink line), acetonitrile (orange line). The absorption at the excited wavelength of each solution was adjusted to 0.05. (emission peaks at 740 nm is attributed to the 2nd order diffracted excitation light.)

In the protic solvents including methanol, **1o** and **1c** did not emit fluorescence, while only **1o** emitted in aprotic solvent, i.e., in hexane, orange fluorescence with a fluorescence quantum yield (Φ_f_) of 0.027. The fluorescent emission spectra of **1o** in several solvents were shown in Figure 4, and λ_max_ of the emission spectra and the fluorescence quantum yields in the solvents are summarized in Table 2. These emission spectra of **1o** were largely red-shifted, indicating the ESIPT property. Generally, the fluorescence quantum yields decreased with increasing the permittivity of the solvents. No fluorescence was observed for methanol and acetonitrile solutions, indicating the suppression of ESIPT, because the solvents were used without dehydration.

**Table 2:** Emission maxima of the fluorescence spectra and the relative fluorescence quantum yields Φ_f_ in several solvents.

solution	λ_max_^a^ / nm	Φ_f_^a^	permittivity / F m^−1^	refractive index [27]

hexane	625	0.027	2.0	1.3727
chloroform	635	0.013	4.8	1.4459
THF	670	0.002	7.5	1.4050
dichloromethane	650	0.006	9.1	1.4242
2-propanol	623	0.004	18	1.3776
methanol	–	–	33	1.3288
acetonitrile	–	–	37	1.3442

^a^λ_ex_ = 370 nm.

**Figure 5:**
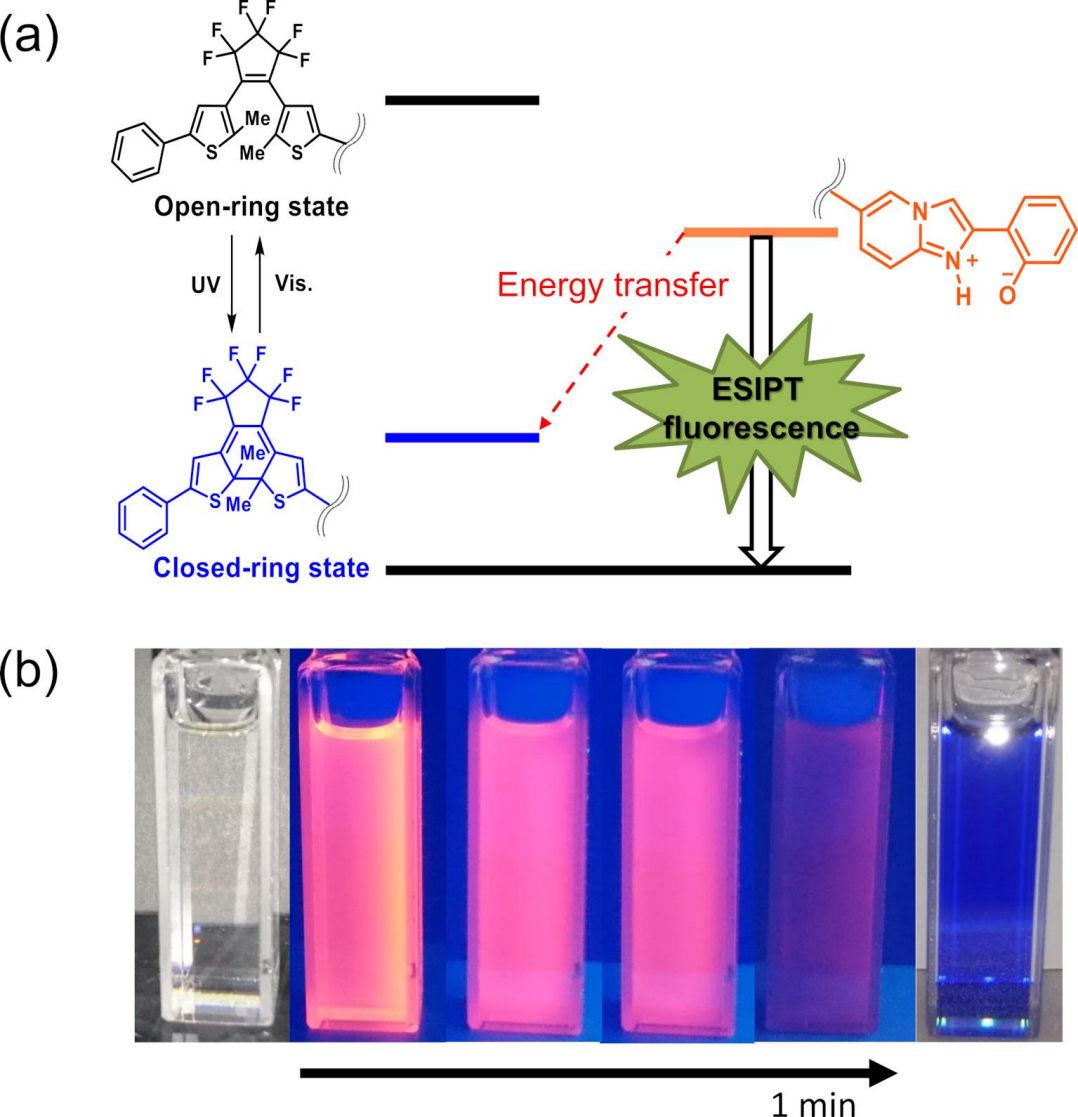
(a) The energy diagram of the ESIPT process of **1**. (b) ESIPT fluorescence quenching upon UV light (λ = 313 nm) irradiation of **1o** in THF solution. The fluorescence is quenched by photo-generated closed-ring isomer **1c**.

The intensity of the fluorescence decreased gradually upon UV light irradiation accompanied with the formation of **1c**, because of excitation energy transfer from the ESIPT moiety to the closed-ring isomer (Figure 5) [4].

The wavelengths of absorption (Table 3) and fluorescence (Table 4) were obtained computationally by using density functional theory (DFT) and time-dependent DFT (TDDFT). The excitation wavelengths as well as the emission wavelength qualitatively agrees with the experimental results. Since compound **1** consists of a diarylethene moiety and an imidazo[1,2-*a*]pyridine moiety, the characteristic of **1** has the combination of these two moieties. As it is expected, the stable structure of the ESIPT is found computationally only in the excited state. (Hereafter, we denote **1o-NH** (**1c-NH**) as the ESIPT state of **1o** (**1c**) and **1o-OH** (**1c-OH**) as the original structure shown in Figure 2 to emphasize the structural difference.) Unexpectedly, however, the optimized structure at the first excited state, **1o-NH** is stable, but **1c-NH** is not (the energy difference between **1c-OH** and **1c-NH** is 11.1 kcal/mol). This is because the first excited state of **1c** is mainly localized on the diarylethene moiety. The HOMO orbital of **1o** is localized on the imidazo[1,2-*a*]pyridine moiety whereas the LUMO orbital is manly localized on the diarylethene moiety. Thus, the proton-transfer in the excited state is favorable at the first excited state of **1o**.

**Table 3:** Excitation energies for **1o** and **1c** in THF.

	excited state	excitation energies (λ)	oscillator strength

**1o**	1	3.38 eV (366 nm)	0.2108
	2	3.48 eV (356 nm)	0.2089
**1c**	1	1.97 eV (630 nm)	0.6744
	2	2.53 eV (490 nm)	0.0308

**Table 4:** Emission energies for **1o** and **1c** in THF.

	excited state	emission energies (λ)	oscillator strength

**1o**	1	1.69 eV (735 nm)	0.0027
	2	2.31 eV (538 nm)	0.3004
**1c**	1	1.40 eV (887 nm)	0.5519
	2	2.23 eV (555 nm)	0.0471

**Figure 6:**
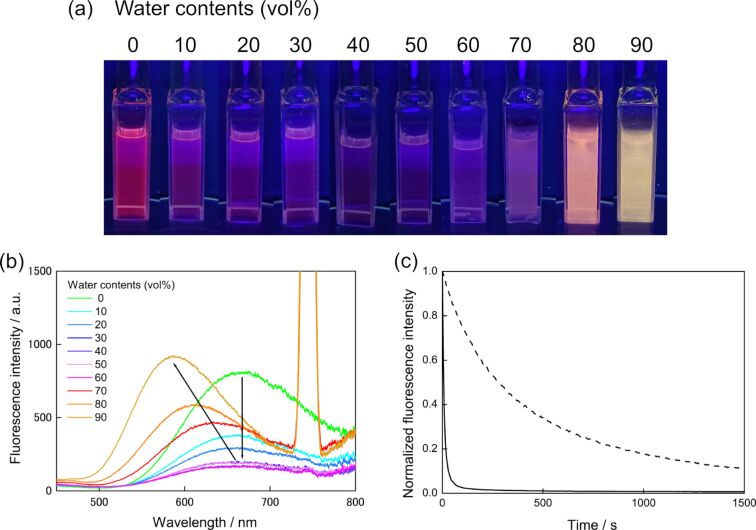
(a) Fluorescence photographs of solutions/suspensions of **1o** (1.2×10^-4^ M) in THF/water mixtures with different water contents under UV light (λ = 365 nm) irradiation. (b) The fluorescence spectra of **1o** solutions (λ_ex_ = 370 nm). (c) The fluorescence quenching of THF 100 vol % (water 0 vol %) solution at 670 nm (broken line) and that of THF/water = 10:90 (v/v) at 585 nm (solid line) upon UV irradiation.

To the THF solution of **1o**, water was gradually added, and the intensities and color changes of the fluorescence were monitored. By adding 10 vol % water the fluorescence was dramatically reduced. However, by the addition of 80 vol %, color of fluorescence changed to orange and the intensity increased (Figure 6a). In the mixture of 90 vol % water and 10 vol % THF the fluorescence band was blue-shifted, and the color changed to yellow, and the intensity was enhanced (Figure 6b). Such a blue shift of ESIPT fluorescence was already reported and it is ascribed to suppression of the stabilization of the excited zwitterionic species through solvent rearrangement and/or further conformational changes of the substrate [19].

The X-ray analysis data of a single crystal of **1o** is shown in Figure S1 and Table S1 in Supporting Information File 1. The distance between two reactive carbon atoms in the thiophene rings was obtained to be 3.534 Å, which is less than 4.2 Å. It indicates the molecule is photoreactive in the crystalline state [28].

**Figure 7:**
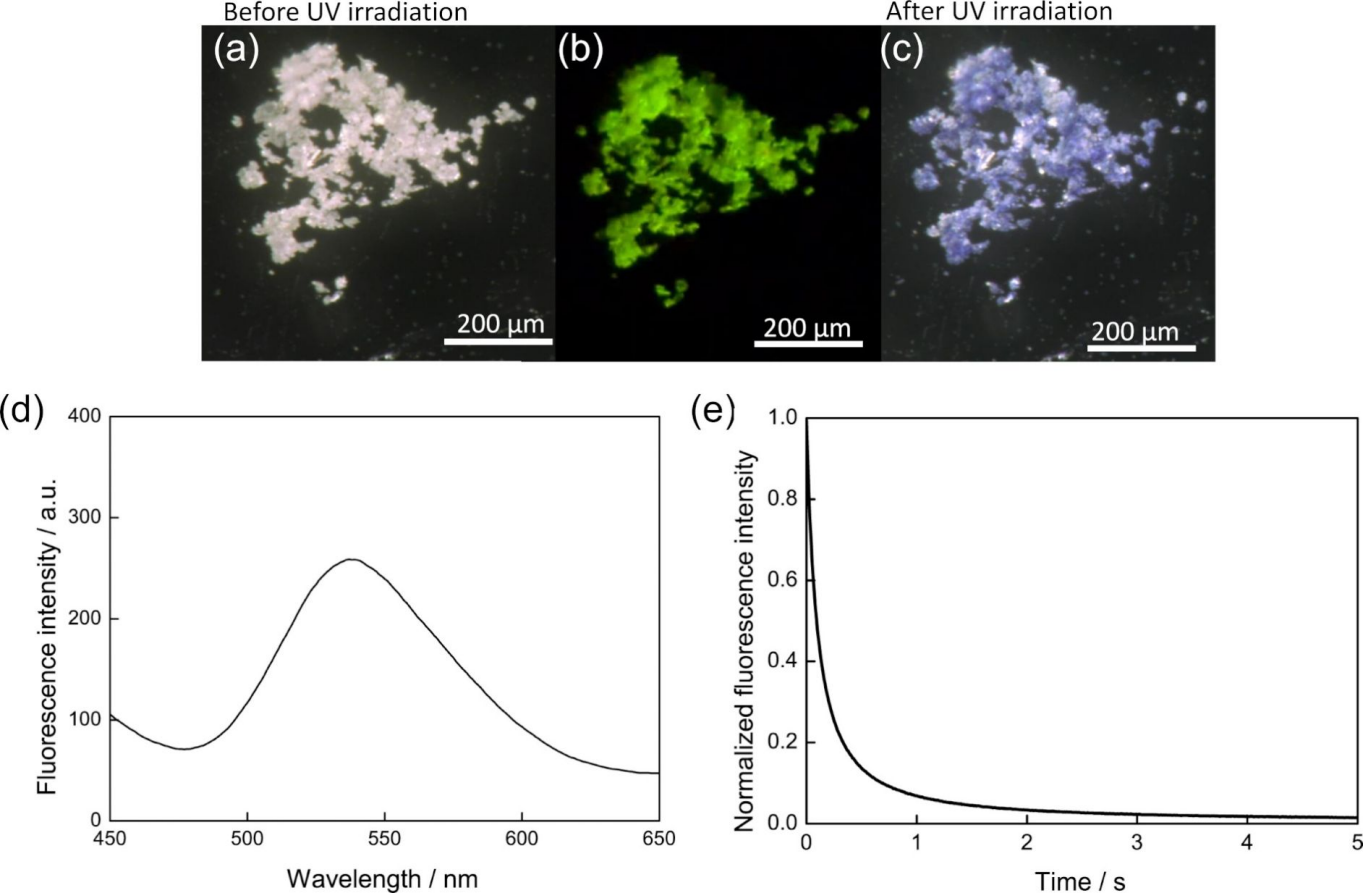
(a) Crystals of **1o** before UV light irradiation, (b) Green fluorescence of **1o** observed under UV light (λ = 365 nm) irradiation, (c) Cyclization proceeded to form **1c** with suppression of the fluorescence, (d) Fluorescence spectra of **1o** in the crystalline state (λ_ex_ = 370 nm, λ_max_ = 537 nm), (e) The fluorescence quenching of **1o** in the crystalline state (λ_ex_ = 370 nm, λ_em_ = 537 nm) upon UV irradiation.

In the crystalline state, **1o** emitted green fluorescence (Φ_f_ = 0.031) as shown in Figure 7b. The color is more blue-shifted color compared with the mixed solution (THF/water = 10:90 (v/v)). The fluorescence is also quenched with the formation of **1c** upon UV light irradiation, indicating turn-off type fluorescent switch (Figure 6c and Figure 7e) [29–30]. The emission from the aggregates quenched much faster than the solution (Figure 6c) (Supporting Information File 2, Movie 1). This is due to a “giant amplification of fluorescence photoswitching” ascribed by a very efficient intermolecular Förster Resonance Energy Transfer (FRET) process between the fluorescent units and the photochromic moieties in their closed form within the aggregated state [29]. The crystal did not show any vapochromism, while a dramatic fluorescent color change from green to pink was observed when chloroform was dropped to the UV light irradiated crystal **1o** (Supporting Information File 3, Movie 2).

The rate of fluorescence quenching (*τ*_1/2_ is less than 0.2 s) in the crystalline state is much faster than those observed in solutions. This is attributed to the degree of condensation which is much higher in the crystalline state. The mechanism of the fast quenching is expected to be related to the amplification of photo-switching of a photochromic organic nanoparticle system as well as the crystalline system reported by Fukaminato and co-workers [29–30]. A detailed study of the amplification will be carried out in the future.

## Conclusion

In summary, we prepared a new diarylethene derivative having an ESIPT functional moiety. It shows the pink fluorescence upon UV light irradiation. Prolonged irradiation with UV light, resulted in the suppressed emission accompanied with a proceeding photocyclization reaction. This is turn-off type fluorescence. The fluorescence was also suppressed in protic and polar solvents because of prohibition of the ESIPT. In the THF/water mixed solvents, when the content of water was increased to 10–60 vol %, the fluorescence was quenched; however, strong fluorescence and blue-shift of emission band was observed above 80 vol % of water content. This is attributable to the AIE effect. The effect was much more remarkable in crystalline state. The emission can be switchable by diarylethene moiety. The effect was also observed in the crystal. The crystal of **1o** emits green fluorescence, and the emission was also suppressed by forming the closed-ring isomer. The crystal shows pink fluorescence once chloroform droplets were dropped on the crystal. The system will be useful for sensors and color indicators.

## Experimental

**General**: Starting materials were commercially available and were used without further purification. Melting points were determined on a Yanaco MP-500D melting point apparatus and are uncorrected. ^1^H (400 MHz), ^13^C (100 MHz) and ^19^F NMR (376 MHz) spectra were recorded on a JEOL JNM-400 spectrometer at ambient temperature. The splitting patterns are designated as follows: s (singlet); d (doublet); dd (double doublet); t (triplet); q (quartet); m (multiplet) and br (broad). Chemical shifts are denoted in δ (ppm) referenced to the residual protic solvent peaks. Coupling constants *J* are denoted in Hz. Mass spectra were recorded on a MALDI-Spiral-TOF-MS mass spectrometer with DCTB (10 mg/mL in CHCl_3_) as a matrix. Absorption and emission spectra were monitored on Hitachi U-4150 spectrophotometer and Hitachi F-7100 fluorescence spectrophotometer, respectively. Fluorescence quantum yields in several solvents were obtained as comparison with that of 9,10-bis(phenylethynyl)anthracene in hexane (Φ_f_ = 1.0) [31]. The luminescence quantum yields of crystalline solids were measured using a JASCO ILF-533 integral sphere attached to a JASCO FP-6600 spectrofluorophotometer. The mixture of microcrystalline compounds (2 mg) and powdered sodium chloride (1 g) were put into a 5 mm quartz cell, which was then placed in the integral sphere. The quantum yield was calculated using an installed software. The solid-state absorption spectrum was obtained by Kubelka–Munk conversion of a diffractive reflectance spectrum of the above mixture measured on a JASCO FP-6600 spectrofluorophotometer equipped with ILF-533 integral sphere. KEYENCE VHX-500, VH-S30, VH-Z20 were used to monitor the crystals. For the UV light irradiation, KEYENCE UV-400, UV-50H (λ *=* 365 nm), Spectronics Corporation Westbury, New York, USA Spectro Line Highest Ultraviolet Intensity Guaranteed (100 V, 40 A, λ = 313 nm) and AS ONE Handy UV Lamp SLUV-4 (λ = 365 nm) were used. For visible light irradiation, a 500W USHIO SX-UI501XQ Xenon lamp attached with Toshiba color filters (Y-48, Y-44, and UV-29) was used.

The Gaussian09 program package [32] was used for geometry optimizations with DFT for ground states and subsequent TDDFT calculations. For the calculation of the fluorescence, the geometry optimizations were performed for the first excited state obtained by TDDFT. The hybrid B3LYP functional [33–35] was adopted to exchange-correlation term of DFT. The gaussian 6-31G(d,p) basis set was adopted to all calculations. As for the solvent effect, polarizable continuum model (PCM) [36] was employed for THF.

X-ray crystallographic analysis for crystal of **1o** was carried out at BL40XU beamline of SPring-8. Si(111) channel cut monochromator was used and the wavelength and the size of X-ray beam were 0.81106 Å and 150 × 150 μm (square), respectively. The diffraction data was collected by oscillation method using EIGER detector at 173 K. The data were corrected for absorption effects by multi-scan method with ABSCOR [37]. The structure was solved by the direct method and refined by the full-matrix least-squares method using the SHELX-2014/7 program. The positions of all hydrogen atoms were calculated geometrically and refined by the riding model. The crystallographic data can be obtained free of charge from The Cambridge Crystallographic Data Center via http://www.ccdc.cam.ac.uk/data_request/cif (CCDC 1920569).

### Synthesis

#### Diarylethene (**2**)

To a 50 mL three neck flask containing 1.15 g (2.40 mmol, 1.0 equiv) of 1-(2-methyl-5-phenylthien-3-yl)-2-(5-chloro-2-methylthien-3-yl)perfluorocyclopentene (**3**) [24] and 15 mL of anhydrous diethyl ether, 2.20 mL (3.52 mmol, 1.5 equiv) of 1.6 N *n*-BuLi in hexane solution was added followed by stirring for 1 h at −10 °C on ice-salt bath under argon gas atmosphere. Then, 0.96 mL (3.58 mmol, 1.5 equiv) of B(OBu)_3_ was added and the temperature of the mixture was allowed to warm to room temperature and stirred for 1 h. After ascertaining the formation of boronic acid by TLC, 5 mL of H_2_O was added, and solvent was removed in vacuo. To a 200 mL three necked flask, the boronic acid, 0.90 g (6.51 mmol, 2.7 equiv) of K_2_CO_3_, 0.72 g (2.37 mmol, 1.0 equiv) of 6-bromo-2-(2’-methoxyphenyl)imidazo[1,2-a]pyridine (**4**) [25], 0.11 g (0.09 mmol, 0.04 equiv) of Pd(PPh_3_)_4_(0), and 80 mL of mixture of 1,4-dioxane/H_2_O (3:1) were added, and refluxed for 16 h. After the reaction was finished, the mixture was cooled down to room temperature. Then the mixture was extracted with 40 mL of diethyl ether for four times. The combined organic layer was washed with 400 mL of water twice and dried over anhydrous sodium sulfate. After filtration, the solvents were removed in vacuo. The residue was purified by silica gel chromatography (eluent: hexane and followed by a mixture of hexane and ethyl acetate (98:2)) to obtain 0.42 g of **2** as a pale-blue oil in 26% yield. The oil was purified with GPC followed by silica gel chromatography (eluent: hexane and followed by a mixture of hexane/diethyl ether (7:3) to obtain 0.31 g (0.47 mmol) of **2** as bluish viscous solid in 19% yield. ^1^H NMR (400 MHz, CDCl_3_, ppm) δ 8.40 (dd, *J* = 7.7, 1.7 Hz, 1H), 8.32 (s, 1H), 8.23 (s, 1H), 7.64 (d, *J* = 9.3 Hz, 1H), 7.54 (d, *J* = 7.8 Hz, 2H), 7.41–7.28 (m, 6H), 7.24 (s, 1H), 7.11 (ddd, *J* = 7.6, 7.4, 1.0 Hz, 1H), 7.02 (d, *J* = 8.2 Hz, 1H), 4.01 (s, 3H), 2.00 (s, 6H); ^13^C NMR (100 MHz, CDCl_3_, ppm) δ 156.9, 143.7, 142.6, 142.2, 141.5, 141.4, 138.4, 133.4, 129.2, 129.2, 129.2, 129.1, 129.0, 128.9, 128.1, 126.1, 125.9, 125.7, 125.6, 123.9, 123.0, 122.5, 122.2, 121.7, 121.2, 119.4, 117.5, 113.2, 111.0, 55.5, 14.7, 14.77; ^19^F NMR (376 MHz, CDCl_3_, ppm) δ −113.1 (s, 2F), −113.3 (s, 2F), −135.0 (s, 2F); HRMS (MALDI–TOF) *m*/*z*: calcd for C_35_H_24_F_6_N_2_OS_2_, 666.1234; found, 666.1229.

#### Diarylethene (**1o**)

To 5 mL of dichloromethane anhydrous solution containing 0.21 g (0.31 mmol, 1.0 equiv) of diarylethene **2**, 1.5 mL (1.5 mmol, 5.0 equiv) of 1.0 M BBr_3_ dichloromethane solution was added on dry-ice/methanol bath (−78 °C) under argon gas atmosphere, and stirred overnight at room temperature. After the reaction was finished, saturated sodium bicarbonate aqueous solution was added to stop the reaction. To the mixture, 70 mL of water was added and the mixture was extracted with 30 mL of chloroform for four times. The combined organic layer was washed with saturated sodium bicarbonate aqueous solution and water, successively, then dried over sodium sulfate anhydrous. After the filtration, solvents were removed in vacuo. The residue was purified by silica gel chromatography (eluent: chloroform) to obtain 0.08 g (0.12 mmol) of **1** as colorless prism shaped crystals in 41% yield. mp 215.0–215.8 °C; ^1^H NMR (400 MHz, CDCl_3_, ppm) δ 12.5 (s, 1H), 8.33 (s, 1H), 7.89 (s, 1H), 7.62 (d, *J* = 9.3 Hz, 1H), 7.59 (dd, *J* = 7.7, 1.5, Hz, 1H), 7.55 (d, *J* = 7.4 Hz, 2H), 7.42 (dd, *J* = 9.3, 1.7, Hz, 1H), 7.40 (t, *J* = 7.4 Hz, 2H), 7.33-7.23 (m, 4H), 7.05 (d, *J* = 7.7 Hz, 1H), 6.90 (t, *J* = 7.7 Hz, 1H), 2.02 (s, 3H), 2.00 (s, 3H); ^13^C NMR (100 MHz, CDCl_3_, ppm) δ 157.4, 146.3, 142.8, 142.6, 142.1, 141.4, 137.7, 133.4, 130.1, 129.2, 129.2, 128.2, 126.3, 125.9, 125.9, 125.8, 125.8, 124.6, 123.7, 122.5, 121.5, 120.7, 119.2, 117.9, 117.0, 116.1, 107.3, 14.7, 14.7; ^19^F NMR (376 MHz, CDCl_3_, ppm) δ −113.1 (s, 2F), −113.3 (s, 2F), −135.0 (s, 2F); HRMS (MALDI–TOF) *m*/*z*: calcd for C_34_H_22_F_6_N_2_OS_2_, 652.1078; found, 652.1072.

## Supporting Information

File 1 X-ray analysis data of a single crystal of **1o**.

File 2 Movie 1, quenching rate dependence on the environmental conditions.

File 3 Movie 2, fluorescent color change of **1o**.
