# Supplementary material for: Aggregation-induced emission effect on turn-off fluorescent switching of a photochromic diarylethene
Source: Beilstein J Org Chem. 2019 Sep 20;15:2204–12. doi: 10.3762/bjoc.15.217 (PMC6774079; doi:10.3762/bjoc.15.217)
Supplement: File 1 — X-ray analysis data of a single crystal of 1o. [file Beilstein_J_Org_Chem-15-2204-s001.pdf]

BEILSTEIN JOURNAL OF ORGANIC CHEMISTRY

Supporting Information

for

Aggregation-induced emission effect on turn-off fluorescent switching of a photochromic diarylethene

Luna Kono, Yuma Nakagawa, Ayako Fujimoto, Ryo Nishimura, Yohei Hattori, Toshiki Mutai, Nobuhiro Yasuda, Kenichi Koizumi, Satoshi Yokojima, Shinichiro Nakamura and Kingo Uchida

Beilstein J. Org. Chem. **2019,** *15,* 2204–2212. doi:10.3762/bjoc.15.217

X-ray analysis data of a single crystal of 1o

License and Terms: This is a supporting information file under the terms of the Creative Commons Attribution License (http://creativecommons.org/licenses/by/4.0). Please note that the reuse, redistribution and reproduction in particular requires that the authors and source are credited.
The license is subject to the *Beilstein Journal of Organic Chemistry* terms and conditions: (https://www.beilstein-journals.org/bjoc)

Table of Contents

1. Figure S1: Molecular structure of **1o** in crystalline state. **S2**

2. Table S1: Single crystalline analysis data of **1o**. **S3**

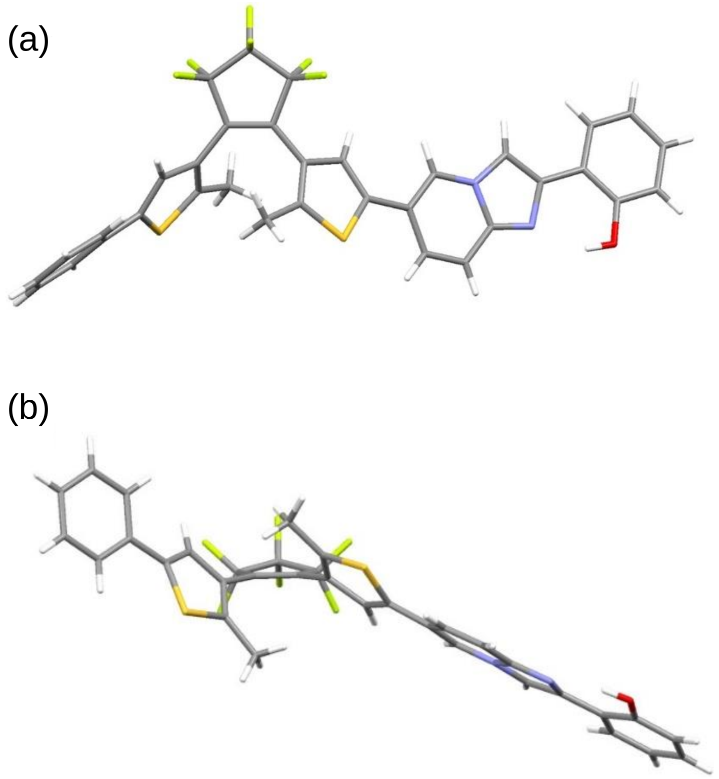

Figure S1: Molecular structure of **1o** in crystalline state, (a) front view, (b) top view.

Table S1: Single crystalline analysis data of **1o.**

	**1o**
Formula	$C_{34}H_{22}F_6N_2OS_2$
formula weight	652.65
T / *K*	173(2)
crystal system	triclinic
space group	P-1
a / Å	8.7814(3)
b / Å	10.9787(4)
c / Å	16.5659(5)
α / °	107.795(2)
β / °	100.339(2)
γ / °	96.313(2)
V / $Å^3$	1472.65(9)
Z	2
R_1 (I > 2s(I))	0.0446
wR_2 (I > 2s(I))	0.0999
R_1 (all data)	0.0806
wR_2 (all data)	0.1457
CCDC No.	1920569
